# Advancing ulcerative colitis therapy: a review of 5-ASA synergy with traditional Chinese medicine and natural bioactive compounds

**DOI:** 10.3389/fphar.2026.1738151

**Published:** 2026-01-29

**Authors:** Weiwei Dong, Hengquan Wu, Tao Liu, Wenting He

**Affiliations:** 1 The Second Hospital & Clinical Medical School, Lanzhou University, Lanzhou, Gansu, China; 2 Gansu Provincial Key Laboratory of Environmental Oncology, Lanzhou, Gansu, China; 3 Digestive System Tumor Prevention and Treatment and Translational Medicine Engineering Innovation Center of Lanzhou University, Lanzhou, Gansu, China

**Keywords:** 5-aminosalicylic acid, combination therapy, naturalbioactive compounds, synergistic effect, traditional Chinese medicine, ulcerative colitis

## Abstract

5-Aminosalicylic acid (5-ASA) is a first-line drug for the treatment of mild to moderate ulcerative colitis (UC), yet its monotherapy efficacy remains limited. In recent years, the combination of 5-ASA with bioactive components of traditional Chinese medicine (TCM) has emerged as an important strategy in UC management. Studies have shown that natural bioactive compounds from TCM (e.g., berberine, curcumin) contribute to synergistic anti-inflammatory and antioxidant effects, as well as enhanced drug-targeted delivery, while TCM formulations (e.g., Gegen Qinlian Decoction, Xilei San) exert holistic multi-target regulation by inhibiting key inflammatory pathways such as nuclear factor-kappa B (NF-κB) and mitogen-activated protein kinase (MAPK), modulating gut microbiota composition, and restoring mucosal barrier function. This combination strategy significantly improves clinical response rates, endoscopic remission, and mucosal healing, without increasing the risk of adverse effects. It reflects a modern treatment philosophy of “Western medicine for primary action, Chinese medicine for synergistic support,” and provides a safe and effective evidence-based approach for optimizing UC therapy.

## 1 Introduction

Ulcerative colitis (UC) is a chronic inflammatory bowel disease characterized by recurrent intestinal inflammation and epithelial injury. Its pathogenesis is closely associated with dysregulated gut immune responses, driven by complex interactions among genetic, environmental, and microbial factors (Ramos and Papadakis, 2019). 5-Aminosalicylic acid (5-ASA) remains a first-line therapy for mild to moderate UC, exerting its effects through local anti-inflammatory actions to induce and maintain remission. However, monotherapy with 5-ASA is limited by suboptimal clinical remission rates (approximately 40%–60%), frequent relapse, reduced efficacy in extensive or severe disease, and inadequate site-specific delivery to inflamed colonic tissues. Long-term use may also lead to adverse effects such as renal impairment (Chand et al., 2025; Veloso et al., 2021). These constraints highlight the exploration of novel therapeutic strategies.

Given these challenges, the integration of TCM with 5-ASA represents a promising therapeutic strategy. With its long-standing history in treating gastrointestinal disorders, TCM emphasizes systemic balance, multi-target interventions, and individualized treatment—principles that align with the multifactorial nature of UC (Tian et al., 2025). The combined approach seeks not only to supplement 5-ASA, but also to engage complementary pathways that may enhance efficacy, promote mucosal healing, and reduce recurrence.

**FIGURE 1:**
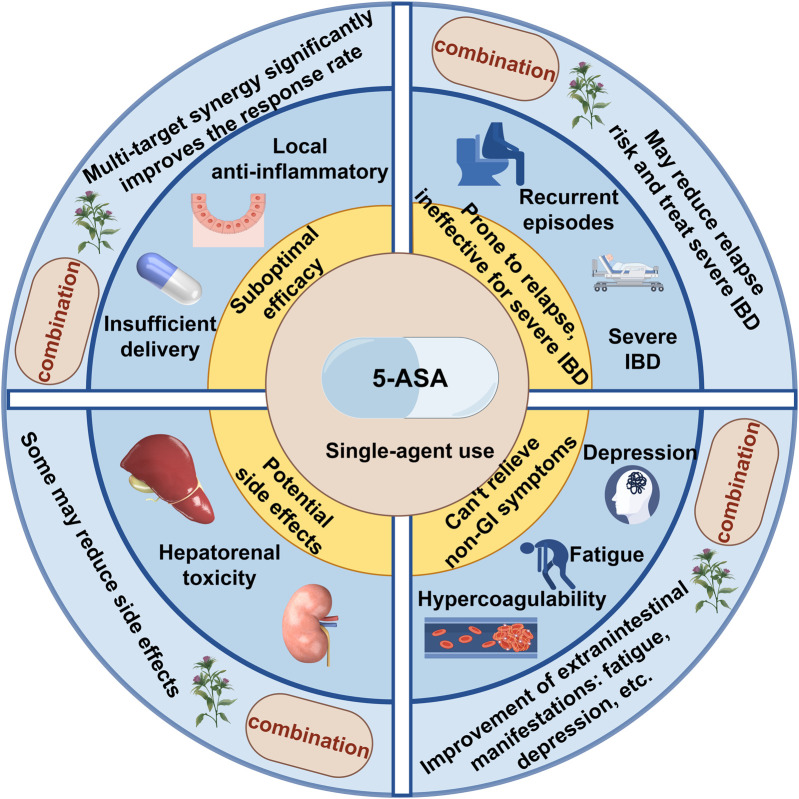
Clinical advantages of combining traditional Chinese medicine and natural bioactive compounds with 5-ASA.

To navigate this evolving field and assess its translational potential, this review first synthesizes the current landscape of research on 5-ASA and TCM combination therapy for UC. As illustrated in Figure 1, this integrated strategy demonstrates multi-target synergistic effects, significantly improving clinical response, endoscopic remission, and mucosal healing rates, while exhibiting a unique “efficacy enhancement and toxicity reduction” profile. We focus on delineating how this integrated approach moves beyond the limitations of conventional monotherapy, explore the emerging evidence supporting its clinical application, and identify key questions that must be addressed to advance its role in modern, precision-oriented UC management.

## 2 Combination therapy with 5-ASA and TCM for UC

### 2.1 Combination of natural bioactive compounds with 5-ASA: multidimensional regulation and delivery enhancement

The combination of natural bioactive compounds with 5-ASA represents a significant advancement in UC therapy, aligning with the emerging trend toward multi-target regulation and synergistic efficacy. This integrated approach capitalizes on the multi-mechanistic actions of natural compounds to complement and enhance the therapeutic effects of 5-ASA, thereby addressing the multifaceted pathology of UC—from acute inflammatory flare-ups to chronic mucosal damage and impaired repair. Consequently, it markedly improves clinical outcomes and disease control. This subsection focuses on natural bioactive compounds that exert direct synergistic effects with 5-ASA at the molecular and tissue levels. These compounds act through relatively defined mechanisms, thereby complementing the pharmacological action of 5-ASA. Table 1 summarizes recent advances (over the past 5 years) in the combined application of natural bioactive compounds and 5-ASA.

**TABLE 1:** Combined application of 5-ASA and natural bioactive compounds.

Compound	Study type	Co-therapy regimen	Mechanisms	Outcomes	Safety	References
Tanshinone IIA	Meta-analysis	Tanshinone IIA + Mesalazine (Dose unspecified)	• Synergistic Anti-inflammation: ↓TNF-α, CRP, MHC-II • Immune Regulation: Inhibits antigen presentation and excessive immune response	Improved overall clinical response rate; enhanced efficacy of mesalazine	No significant increase in adverse drug reactions	Chen et al. (2024)
Berberine	Meta-analysis	Berberine: 0.2–2 g, 3 times/day + 5-ASA: 0.5–1g, 3 times/day (Oral or Rectal)	• Synergistic Anti-inflammation: ↓IL-6, IL-8, TNF-α; ↑IL-10 • Immune Regulation: ↑CD4+ T cells, modulates CD4+/CD8+ ratio; activates IL-4/STAT6 pathway, promotes M2 macrophage polarization • Microbiota Modulation: ↓Bacteroidetes/Proteobacteria, ↑Firmicutes • Mucosal Repair: Improves intestinal mucosal damage	Improved clinical response rate; reduced Baron endoscopic score and disease activity index (DAI)	No significant increase in adverse reactions when combined with 5-ASA; favorable safety profile	Li et al. (2024)
Curcumin	Meta-analysis	Curcumin: 0.1–10 g/day + mesalamine (oral)	• Synergistic Anti-inflammation: Inhibits NF-κB signaling pathway; ↓TNF-α, IL-1β • Immune Regulation: Modulates Th1/Th2 cell balance	Clinical response rate nearly three times higher than placebo when combined with mesalamine	Minimal side effects with combination therapy	Chandan et al. (2020)
Cannabidiol	Animal study	Cannabidiol: 10 mg/kg/day + Olsalazine: 50 mg/kg/day (Oral, mice)	• Synergistic Anti-inflammation: Inhibits NF-κB pathway; ↓TNF-α, IL-6, IL-1β, MPO activity • Immune Regulation: Modulates CB1/CB2 and GPR35 receptors • Barrier Repair: Maintains epithelial integrity	Enhanced therapeutic effects of olsalazine and cyclosporine in colitis; improved symptoms and pathological damage	Low dose (10 mg/kg) combined use did not induce hepatorenal toxicity; reduces side effects	Thapa et al. (2025)
Arbutin	Animal study	Arbutin: 250 mg/kg/day + Mesalazine: 100 mg/kg/day (Oral, rats)	• Synergistic Anti-inflammation: Inhibits NF-κB pathway; ↓TNF-α, IL-6, IL-1β • Antioxidant: ↓MDA, MPO; ↑GPx, SOD • Mucosal Protection: Reduces mucosal loss	Improved oxidative stress and inflammation; significantly reduced mucosal damage when combined with mesalazine	No adverse effects reported	Alemdar et al. (2024)
Glucosamine	Animal study	D-Glucosamine: 300 mg/kg/day + 5-ASA: 25.5 mg/kg/day (Oral, rats)	• Synergistic Anti-inflammation: ↓IL-1β, TNF-α • Antioxidant: ↓MDA; ↑GSH • Multi-target Action: Synergizes with 5-ASA	Improved inflammation and restored colonic tissue structure	No major adverse reactions reported	Roy et al. (2023)
Ganoderma lucidum	Animal study	Ganoderma lucidum: 100 mg/kg/day (Oral) + Mesalazine: 200 mg/kg/day (Rectal, Rats)	• Synergistic Anti-inflammation: Inhibits NF-κB pathway; ↓TNF-α, IL-6 • Antioxidant: ↓MDA; ↑SOD	Significantly reduces intestinal inflammation and severity of mucosal damage	Good safety profile in rat models	Özden et al. (2022)
Chitosan	Animal study	Chitosan: 30 mg/kg/day + 5-ASA: 30 mg/kg/day (Rectal, mice)	• Synergistic Anti-inflammation: ↓TNF-α, IL-6 • Mucosal Protection; Delivery Enhancement	Combination with 5-ASA is more effective than 5-ASA alone in reducing colon inflammation and tissue damage	Good safety profile in rat models	Jhundoo et al. (2020)
Hyaluronic acid	Animal study	Hyaluronic Acid: 15–30 mg/kg/day + 5-ASA: 30 mg/kg/day (Rectal, mice)	• Mucosal Protection & Repair: Forms a hydrated protective layer; promotes epithelial cell migration, proliferation, and differentiation • Synergistic Anti-inflammation: Inhibits NF-κB activation; ↓TNF-α, IL-6, IL-1β; neutralizes ROS • Complementary Mechanism: High mucoadhesion prolongs 5-ASA retention, forming a sustained release system	Significantly reduces clinical and histological scores; combination with 5-ASA is superior to 5-ASA monotherapy in severe colitis	No adverse effects reported	Jhundoo et al. (2021a)
Acacia and guar gum	Animal study	Acacia: 300 mg/kg/day + 5-ASA: 30 mg/kg/day (Rectal, mice)	• Synergistic Anti-inflammation: Inhibits MPO and NF-κB activity • Mucosal Protection & Repair: Binds to mucin via H-bonds and electrostatic interactions, forming an additional gel protective layer on the mucosal layer • Microbiota & Metabolic Modulation: ↑SCFAs (acetate, propionate, butyrate); restores gut microbiota	Combination with 5-ASA significantly improves colitis pathological indices; Acacia + 5-ASA showed the best effect	No adverse effects reported	Jhundoo et al. (2021b)
Arabinoxylan	Animal study	Arabinoxylan: 250 mg/kg/day + 5-ASA: 150 mg/kg/day (Oral, mice)	• Synergistic Anti-inflammation: ↓IL-1β, IL-6, TNF-α, serum LPS • Microbiota Modulation: ↑*Lactobacillus*, Lachnospiraceae; ↓Escherichia-Shigella, *Helicobacter* • Metabolic Modulation: ↑SCFAs (butyrate) • Barrier Repair: ↑MUC-2, Occludin, ZO-1, Claudin-1	Improves disease activity index, restores colon length, promotes mucosal healing; efficacy comparable to 5-ASA, superior in boosting SCFAs and repairing mucus barrier	No adverse effects reported	Huang et al. (2025)
Lactulose	Animal study	Lactulose: 1,000 mg/kg/day + Mesalazine: 400 mg/kg/day (Oral, rats)	• Microbiota Modulation: ↑Muribaculaceae, Prevotellaceae; ↓Proteobacteria, *Clostridium* • Metabolic Modulation: ↑SCFAs (acetate, propionate, butyrate) • Barrier Repair: Improves microvilli structure, enhances tight junctions, ↑goblet cells and mucus secretion • Synergistic Anti-inflammation: ↓IL-6, TNF-α, Hs-CRP; inhibits TLR/NF-κB pathway • Cytoprotection: Repairs mitochondrial function, reduces oxidative stress	Improves bloody stool and diarrhea, increases body weight; alleviates colon inflammation pathology; shows additive therapeutic effect with mesalazine	Low dose (10 g/d human equivalent) has almost no GI adverse effects	Cui et al. (2025)

Abbreviations: ↑, increase; ↓, decrease; TNF-α, tumor necrosis factor-alpha; CRP, C-reactive protein; MHC-II, major histocompatibility complex class II; IL, interleukin; STAT6, signal transducer and activator of transcription 6; NF-κB, nuclear factor-kappa B; MPO, myeloperoxidase; CB1/CB2, cannabinoid receptors 1/2; GPR35, G protein-coupled receptor 35; MDA, malondialdehyde; GPx, glutathione peroxidase; SOD, superoxide dismutase; GSH, glutathione; ROS, reactive oxygen species; SCFAs, short-chain fatty acids; LPS, lipopolysaccharide; MUC-2, mucin 2; ZO-1, zonula occludens-1; Hs-CRP, high-sensitivity C-reactive protein; TLR, Toll-like receptor; DAI, disease activity index.

During active disease, UC is characterized by excessive activation of immune pathways and a surge in pro-inflammatory mediators. Natural bioactive compounds synergize with 5-ASA to potently suppress these acute inflammatory responses through multiple, complementary mechanisms (Tang et al., 2025). In terms of immune and inflammatory regulation, several natural active ingredients demonstrates multi-target synergistic effects with 5-ASA. Meta-analyses based on randomized controlled trials (RCTs) have provided evidence that tanshinone IIA enhances the anti-inflammatory efficacy of 5-ASA by inhibiting major histocompatibility complex class II (MHC-II)-mediated antigen presentation and reducing levels of tumor necrosis factor-alpha (TNF-α) and C-reactive protein (CRP) (Chen et al., 2024). Berberine promotes M2 macrophage polarization through activation of the interleukin-4/signal transducer and activator of transcription 6 (IL-4/STAT6) pathway. In parallel, it modulates the CD4^+^/CD8^+^ T-cell balance, which is associated with significant reductions in the Baron endoscopic score and disease activity index (DAI) (Li et al., 2024). Curcumin not only exerts synergistic anti-inflammatory effects by inhibiting the NF-κB signaling pathway but also regulates the T helper 1/T helper 2 (Th1/Th2) cell balance, leading to a significant improvement in clinical response rates (Chandan et al., 2020). In animal models, cannabidiol demonstrates multi-receptor regulatory potential by acting on cannabinoid receptor 1/cannabinoid receptor 2 (CB1/CB2) and G protein-coupled receptor 35 (GPR35). Through these receptors, cannabidiol synergistically inhibits NF-κB signaling, reduces the release of pro-inflammatory cytokines such as IL-6 and IL-1β, and enhances the therapeutic efficacy of olsalazine and cyclosporine (Thapa et al., 2025).

Concurrently, these compounds combat oxidative stress, a key driver of tissue damage in acute inflammation (Muro et al., 2024). Animal studies have indicated that arbutin inhibits the NF-κB pathway, reduces the expression of inflammatory cytokines such as TNF-α, IL-6, and IL-1β, and enhances the activity of antioxidant enzymes including glutathione peroxidase (GPx) and superoxide dismutase (SOD), thereby effectively alleviating oxidative damage and mucosal tissue injury (Alemdar et al., 2024). D-Glucosamine has been shown to decrease IL-1β and TNF-α levels while increasing glutathione (GSH) content, synergizing with 5-ASA to ameliorate colonic histopathological structure (Roy et al., 2023). Similarly, Ganoderma lucidum extract exerts its effects through dual antioxidant and anti-inflammatory mechanisms, involving reduction of malondialdehyde (MDA), elevation of SOD activity, and suppression of TNF-α and IL-6 expression, leading to marked attenuation of intestinal mucosal damage (Özden et al., 2022).

Following the suppression of acute inflammation, successful UC therapy necessitates active promotion of mucosal healing and restoration of intestinal homeostasis (Otte et al., 2023). The combination therapy excels in this phase by enhancing barrier function and modulating the gut microenvironment. Certain natural polymers enhance drug delivery and mucosal retention. Chitosan acts as both a mucosal protective effects and a delivery-enhancing carrier, improving 5-ASA delivery and anti-inflammation efficacy (Jhundoo et al., 2020). Hyaluronic acid, through CD44 targeting capability and strong mucosal adhesiveness, prolongs 5-ASA retention at inflammatory sites, enables sustained release, and promotes epithelial cell migration and differentiation. Consequently, the combined treatment exhibits significantly enhanced efficacy over monotherapy in severe colitis models (Jhundoo et al., 2021a). Arabic gum and guar gum form protective gel layer via mucin interactions, enhancing barrier function. Notably, the combination of Arabic gum with 5-ASA achieves therapeutic effects nearly comparable to those observed in healthy control groups (Jhundoo et al., 2021b).

Natural components also modulate the gut microbiota and metabolism microenvironment. Acacia and guar gums exert synergistic therapeutic effects by promoting the production of short-chain fatty acids (SCFAs; e.g., acetate, propionate, butyrate) and restoring the gut microbiota structure (Jhundoo et al., 2021b). Arabinoxylan markedly enhances the abundance of beneficial bacteria, including *Lactobacillus* and Lachnospiraceae_NK4A136_group, along with increased butyrate production. Concomitantly, it upregulates mucin-2 (MUC-2) and key tight junction proteins such as Occludin, zonula occludens-1 (ZO-1), and Claudin-1. Collectively, these changes translate into a more pronounced improvement in mucosal barrier repair than that achieved with 5-ASA alone (Huang et al., 2025). Lactulose enhances the levels of beneficial bacteria including Lachnospiraceae and Prevotellaceae, promotes the generation of SCFAs such as acetate, propionate, and butyrate, and simultaneously inhibits the Toll-like receptor/NF-κB (TLR/NF-κB) pathway, thereby alleviating intestinal inflammation and pathological damage through coordinated actions on microbiota remodeling, metabolite-mediated effects, and immunomodulation (Cui et al., 2025). Notably, by promoting beneficial bacteria and suppressing potential pathogens, these natural components not only directly exert anti-inflammatory and barrier-protective effects but may also reshape the microbial ecology to reduce the abundance of bacteria with 5-ASA-metabolizing activity (e.g., some Proteobacteria), thereby protecting 5-ASA from microbial enzymatic inactivation and enhancing the synergistic effect of combination therapy via improved drug bioavailability (Mehta et al., 2023).

**FIGURE 2:**
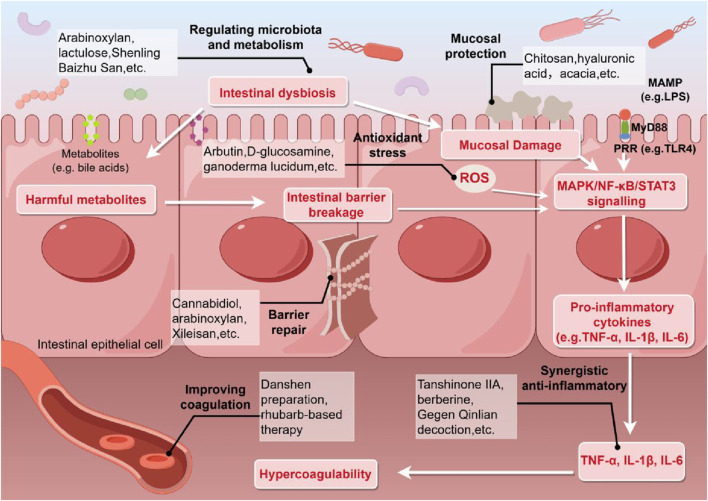
Synergistic mechanisms of combining TCM and natural bioactive compounds with 5-ASA in the treatment of UC.

The combination of natural bioactive compounds with 5-ASA offers a novel therapeutic strategy for ulcerative colitis through a stage-specific, multi-target synergistic approach.These complementary mechanisms—spanning acute immunomodulation, antioxidative damage, followed by active mucosal repair, and microbiota-metabolic regulation—are visually integrated in Figure 2, which provides a comprehensive overview of their temporally coordinated actions. This approach not only produces synergistic effects across these levels but also enhances drug delivery efficiency and mucosal retention through the use of natural polymeric materials, thereby comprehensively improving the efficacy of 5-ASA (Jhundoo et al., 2020; Jhundoo et al., 2021a; Jhundoo et al., 2021b). Studies have demonstrated that such integrated treatment significantly improves clinical response rates and quality of mucosal healing. In particular, the combination of hyaluronic acid and 5-ASA has shown remarkable therapeutic benefits in severe colitis (Jhundoo et al., 2021a). Furthermore, combination therapy exhibits a favorable safety profile, with certain natural compounds such as curcumin and cannabidiol demonstrating the potential to enhance efficacy while reducing toxicity (Chandan et al., 2020; Thapa et al., 2025), opening new avenues for the systematic treatment of ulcerative colitis. Importantly, the upstream molecular basis underlying the synergistic actions of many promising natural compounds remains insufficiently defined. Beyond their well-recognized antioxidant and anti-inflammatory effects, compounds such as tanshinone IIA, berberine, and curcumin are likely to interact with 5-ASA through modulation of key upstream signaling pathways, including MAPK/ERK and PI3K/AKT (Zhang et al., 2023; Zhou et al., 2025; Patnaik et al., 2025). Accordingly, systematically delineating the temporal and spatial coordination between these natural agents and 5-ASA along these pathways represents a critical direction and a major opportunity for future mechanistic studies.

### 2.2 Combination of TCM formulations and 5-ASA: holistic multi-target regulation

The combination of TCM formulations with 5-ASA demonstrates multidimensional synergistic advantages in clinical efficacy, mechanisms of action, and safety, significantly expanding the scope of disease management beyond conventional 5-ASA monotherapy. In contrast to single bioactive compounds, traditional Chinese medicine formulations are characterized by multi-component and system-level regulatory effects. This subsection emphasizes the broader and more holistic mechanisms by which TCM formulations synergize with 5-ASA. Table 2 systematically summarizes key research advances in the combined application of TCM formulations and 5-ASA over the past 5 years.

**TABLE 2:** Combined application of 5-ASA and traditional Chinese medicine.

TCM	Study type	Co-therapy regimen	Mechanisms	Outcomes	Safety	References
Gegen Qinlian decoction	Meta-analysis + Trial Sequential analysis	Gegen Qinlian decoction + Mesalazine (Dose unspecified)	• Synergistic Anti-inflammation: Inhibits TLR, TNF, MAPK pathways • Multi-target Regulation: Modulates STAT3, IL-6, etc. • Barrier Repair, Microbiota Modulation, Antioxidant	↑ Clinical response rate by 22%; Improved intestinal barrier function	↓Incidence of adverse reactions by 41%	Chen et al. (2025)
Xileisan	Meta-analysis + Trial Sequential analysis	Xileisan: 1.0–2.0 g/d (Oral) + Mesalazine: 1.0–4.0 g/d (Oral)	• Synergistic Anti-inflammation: ↓TNF-α, IL-6 • Barrier Repair: ↑Occludin/Claudin-1 • Immune Regulation: ↑sIgA, ↓β-defensin • Improved Bleeding: ↓eNOS, ↓VEGF	↑ Clinical response rate by 22%; ↑ Mucosal improvement rate by 25%	Did not increase gastrointestinal adverse reactions	Yang et al. (2024)
Danshen preparation	Meta-analysis	DanShen Injection: 20 mL/day (Ivgtt) + Mesalazine: 3.0–4.0 g/day (Oral); Tanshinone Capsules: 3.0 g/day (Oral)/Tanshinone IIA Injection: 80 mg/day (Ivgtt) + Mesalazine: 2.4–4.0 g/day (Oral)	• Improved Coagulation: ↓PLT, ↑MPV (Mean Platelet Volume) • Synergistic Anti-inflammation: ↓CRP, TNF-α, IL-6, IL-8 • Promoting Blood Circulation: Improved intestinal mucosal microcirculation	↑ Clinical response rate by 16%; Improved coagulation function	Favorable safety profile	Zhang W et al. (2024)
Rhubarb-based therapy	Meta-analysis	Rhubarb-based medicinal formulas + 5-ASA or SASP (Dose unspecified)	• Improved Microcirculation: ↓Platelets (PLT), ↓P-selectin, ↓Thromboxane • Synergistic Anti-inflammation: ↓TNF-α, IL-1β, IL-6, CRP; ↑IL-10 • Endothelial Protection: Antagonizes MMP-9, protects tight junctions	Improved hypercoagulable state, increased clinical efficacy; earlier intervention (6–9 weeks) showed better results	Favorable safety profile	Li Y et al. (2022)
Jianpi Qingchang decoction	RCT	Jianpi Qingchang Decoction: twice daily (oral) + Mesalazine: 3.0 g/day (1 g, three times daily, oral)	• Gut Microbiota-Metabolite Axis Regulation: ↓Erysipelotrichaceae; ↓Harmful metabolites (e.g., L-glutamate) • Spleen Invigoration & Anti-fatigue: Improved energy metabolism	Significantly alleviated UC symptoms and fatigue; reduced relapse risk	Favorable safety profile	Liu et al. (2024)
Qing-Chang-Hua-Shi granule	RCT	Qing-Chang-Hua-Shi granule: 125 g/day (Oral) + Mesalazine: 4 g/day (Oral)	• Multi-pathway Anti-inflammation: Inhibits MAPK/NF-κB/ERK pathways • Reduced Oxidative Stress	Increased clinical remission rate and mucosal healing rate	Favorable safety profile	Shen et al. (2021)
Shenling Baizhu San	Prospective cohort study	Shenling Baizhu San: 18 g/day (6 g, three times daily, Oral) + Mesalamine: 4 g/day (Oral)	• Microbiota Remodeling: ↑*Bacteroides*, Blautia, Bifidobacterium, *Lactobacillus* • Metabolic Modulation: ↑Beneficial metabolites (IPA, IAA) • Pathway Activation: Activates AhR/PXR pathways • Enhanced Barrier Function	Increased clinical remission rate, promoted mucosal healing	Favorable safety profile	Jiao et al. (2022)
Qingchang Yuyang decoction	Retrospective cohort study	Qingchang Yuyang decoction: 200 mL/day (oral, divided into morning and evening doses) + Mesalazine sustained-release	• Immune Regulation: ↑IL-10, ↓IL-8, TNF-α • Restored Gut Microbiota Balance	Improved efficacy, alleviated clinical symptoms, reduced inflammation	Lower complication rate	Wang J et al. (2025)
Wuling powder	Animal study	Wuling Powder: 0.5–2.0 g/kg/day + Mesalazine: 0.25 g/kg/day (Oral, mice)	• Brain-Gut Axis Modulation: BDNF/TrkB/sortilin and proBDNF/p75NTR signaling pathways • Multi-component Anti-inflammatory & Antidepressant Effects	Simultaneously improved intestinal inflammation and depressive-like behaviors	No significant adverse reactions observed	Wang JJ et al. (2025)

Abbreviations: ↑, increase; ↓, decrease; TLR, Toll-like receptor; TNF, tumor necrosis factor; MAPK, mitogen-activated protein kinase; STAT3, signal transducer and activator of transcription 3; IL, interleukin; sIgA, secretory immunoglobulin A; eNOS, endothelial nitric oxide synthase; VEGF, vascular endothelial growth factor; PLT, platelets; MPV, mean platelet volume; CRP, C-reactive protein; MMP-9, matrix metalloproteinase-9; IPA, indole-3-propionic acid; IAA, indole-3-acetic acid; AhR, aryl hydrocarbon receptor; PXR, pregnane X receptor; BDNF, brain-derived neurotrophic factor; TrkB, tropomyosin receptor kinase B; p75NTR, p75 neurotrophin receptor.

In terms of clinical efficacy, the combination therapy demonstrated comprehensive and significant improvements. High-level evidence from multiple meta-analyses of RCTs indicates that the combination of Chinese herbal formulas with 5-ASA significantly enhances clinical response rates, mucosal healing rates, and clinical remission rates. For instance, Gegen Qinlian Decoction increased the clinical response rate by 22% (Chen et al., 2025), while Xileisan not only improved the clinical response rate by 22% but also significantly increased the mucosal improvement rate by 25%, effectively alleviating core symptoms such as diarrhea and bloody stool (Yang et al., 2024). Notably, the combination therapy also exhibited systemic regulatory effects on extraintestinal manifestations. For example, Salvia-based and rhubarb-based formulations improved microcirculation, modulated platelet function, and ameliorated hypercoagulability (Zhang W et al., 2024; Li X et al., 2022). Jianpi Qingchang Decoction improved energy metabolism and significantly relieved fatigue (Liu et al., 2024); and Wuling Powder concurrently alleviated intestinal inflammation and depression-like behaviors through brain–gut axis modulation, highlighting the holistic regulatory characteristics of traditional Chinese medicine (Wang JJ et al., 2025).

At the mechanistic level, the combined application of Chinese herbal formulas and 5-ASA achieved multi-dimensional, multi-target synergistic regulation. Gegen Qinlian Decoction and Qingchang Huashi Granule inhibited key inflammatory pathways such as NF-κB, MAPK, and TLR, synergistically downregulating pro-inflammatory cytokines including TNF-α, IL-6, and IL-1β, thereby enhancing anti-inflammatory effects (Chen et al., 2025; Shen et al., 2021). In terms of barrier repair, Xilei San upregulated the expression of tight junction proteins such as Occludin and Claudin-1, while rhubarb-based formulations protected endothelial barrier integrity by antagonizing MMP-9, collectively promoting the restoration of intestinal mucosal barrier function (Yang et al., 2024; Li Y et al., 2022). Regarding immunomodulation, Xileisan also promoted the secretion of secretory immunoglobulin A (sIgA), contributing to the maintenance of immune homeostasis (Yang et al., 2024). Furthermore, formulations such as Jianpi Qingchang Decoction and Shenling Baizhu Powder remodeled the gut microbiota structure by increasing the abundance of beneficial bacteria such as *Bacteroides* and *Lactobacillus*. Shenling Baizhu San additionally promotes the production of beneficial metabolites, including indolepropionic acid and indoleacetic acid. It also activates protective signaling pathways such as AhR/PXR, thereby exerting synergistic regulatory effects at the microbiota–metabolism interface (Liu et al., 2024; Jiao et al., 2022). On the other hand, Salvia-based and rhubarb-based formulations improved intestinal microcirculation and alleviated hypercoagulability by modulating platelet count, P-selectin, and thromboxane levels (Zhang W et al., 2024; Li X et al., 2022); Wuling Powder mediated brain–gut interactions via the BDNF/TrkB/sortilin and proBDNF/p75NTR signaling pathways, simultaneously improving intestinal inflammation and emotional disorders, reflecting the holistic concept of “simultaneous regulation of the intestine and brain” in traditional Chinese medicine (Wang JJ et al., 2025).

Regarding safety, the combination therapy generally exhibited a favorable tolerance profile. Multiple studies indicated that the combined medication did not increase adverse reactions. Moreover, Gegen Qinlian Decoction reduced the incidence of adverse reactions by 41% (Chen et al., 2025), and the complication rate in the Qingchang Yuyang Decoction group was lower than that in the control group (Wang J et al., 2025). These results suggest that the combination of Chinese herbal formulas with 5-ASA not only did not increase safety risks but may also possess the potential to reduce toxicity and enhance efficacy.

Compared with 5-ASA monotherapy, the combination strategy with Chinese herbal formulas not only enhanced anti-inflammatory effects but also addressed the limitations of monotherapy across multiple aspects including immunomodulation, mucosal barrier repair, microbiota–metabolite balance, microcirculation improvement, and brain–gut axis regulation, achieving a transition from local anti-inflammation to systemic holistic regulation (Figure 2). Furthermore, a meta-analysis based on RCTs provided high-level evidence indicating that this combination regimen significantly improved clinical efficacy while effectively reducing the recurrence rate and incidence of adverse reactions in ulcerative colitis patients, highlighting its notable advantage in “toxicity reduction” (Zhang M et al., 2024). The multi-component, multi-target nature of Chinese herbal formulas may further contribute to this holistic benefit by modulating upstream signaling networks. For example, Gegen Qinlian Decoction and rhubarb-based formulations have been reported to ameliorate intestinal inflammation through integrated regulation of pathways such as PI3K/AKT/NF-κB (Chen et al., 2023; Li Y et al., 2022). In summary, the combination of Chinese herbal formulas with 5-ASA operates via multi-target mechanisms and demonstrates comprehensive therapeutic value for the long-term management of ulcerative colitis.

The clinical benefits outlined in Figure 1 are further corroborated by accumulating data. Gegen Qinlian Decoction with 5-ASA increases clinical response rates by approximately 22%, while Xilei San improves mucosal healing by about 25% (Chen et al., 2025; Yang et al., 2024). Combination therapy also shows significantly lower adverse reaction incidence compared to 5-ASA alone (Chen et al., 2025). Beyond intestinal outcomes, TCM formulations exhibit broader regulatory effects Salvia- and rhubarb-based preparations ameliorate intestinal microcirculation and hypercoagulability (Zhang W et al., 2024; Li X et al., 2022), while Wuling Powder modulates both intestinal inflammation and depressive-like behaviors via the brain-gut axis (Wang JJ et al., 2025). Natural carriers such as hyaluronic acid further enhance local drug retention and bioavailability, improving outcomes in severe colitis models (Jhundoo et al., 2021a). These findings collectively validate the therapeutic advantages of integrating TCM and natural bioactive compounds with 5-ASA for the long-term management of ulcerative colitis.

## 3 Challenges and prospects in clinical translation

Although combination therapy with 5-ASA and natural active ingredients from TCM has demonstrated promising synergistic effects and therapeutic potential in both basic and preclinical studies of UC, its translation into large-scale clinical applications still faces multiple challenges. Systematic efforts are required to advance research on mechanistic understanding, personalized treatment, safety evaluation, and delivery systems.

Mechanistic research requires greater depth and systematicity. While current research on combination therapies predominantly focuses on downstream therapeutic biomarkers—such as pro-inflammatory cytokines (e.g., TNF-α, IL-1β, IL-6), oxidative stress markers (e.g., MDA, SOD), and mucosal healing indicators (e.g., MUC-2, tight junction proteins)—investigations into upstream signaling pathways (e.g., Notch, MAPK/ERK) remain preliminary. This creates a significant gap in the depth and systematic rigor of mechanistic understanding. The inherent multi-component, multi-target nature of TCM formulations and natural compounds further complicates this challenge. Although studies have begun to address key pathways like NF-κB and MAPK, critical questions persist: the interactions among different bioactive components, their integrated effects within broader regulatory networks, and their pharmacokinetic interplay with 5-ASA. For instance, the specific manner in which flavonoids and alkaloids in Gegen Qinlian Decoction synergistically modulate the intestinal immune microenvironment and complement 5-ASA pharmacodynamically and pharmacokinetically warrants deeper exploration. Therefore, future research must prioritize elucidating the dynamic changes in these upstream signaling pathways during combination therapy. A systematic approach integrating advanced methodologies such as metabolomics, network pharmacology, and artificial intelligence is essential. Constructing a multidimensional “component-target-pathway-disease” network will be key to unraveling the synergistic mechanisms between TCM and 5-ASA (Liao et al., 2025).

Second, the development of a personalized treatment system is still in its infancy. TCM emphasizes “treatment based on syndrome differentiation,” yet there is a lack of precise diagnostic criteria that integrate modern medical classifications with TCM syndrome types. The clinical study on Jianpi Qingchang Decoction for UC with spleen deficiency and dampness-heat syndrome (Liu et al., 2024) provides a preliminary example. Future work should focus on establishing an integrated classification system that combines microbiome, metabolomic, and immunomic profiles with TCM syndromes, developing biomarker-based personalized medication strategies, and constructing efficacy prediction models to enable pre-treatment response assessment and advance precision medicine in UC (Verstockt et al., 2021).

Third, given the variable efficacy and specific risks associated with combination therapies, rigorous safety evaluation is essential. Although short-term tolerability appears favorable, comprehensive long-term assessment remains necessary. Although high-quality clinical studies directly reporting combination therapies as “ineffective” or “harmful” are uncommon, inconsistencies persist in the existing evidence. These mainly involve nonsignificant differences in secondary endpoints, insignificant results in subgroup analyses, and potential pharmacological risks. For example, IL-6 levels showed no significant change in studies combining tanshinone IIA (Chen et al., 2024); negative results have been reported in some trials of curcumin combination therapy (Chandan et al., 2020); and no differences in oxidative stress markers were observed in studies on Gegen Qinlian Decoction (Chen et al., 2025). Moreover, several bioactive compounds pose identifiable risks. For examople, berberine may inhibit CYP450 enzymes and affect concomitant drugs (Bathaei et al., 2025). Cannabidiol shares metabolic pathways with several neuroactive agents (Chesney et al., 2020). Meanwhile, curcumin demonstrates complex, dose-dependent effects on platelet function, exhibiting antiplatelet activity at higher doses while potentially potentiating platelet apoptosis at lower concentrations (Rukoyatkina et al., 2021). Notably, gut microbiota can metabolize 5-ASA, suggesting that certain TCM components might inadvertently reduce its bioavailability (Mehta et al., 2023). Future efforts should therefore systematically map interaction profiles, evaluate microbiota-mediated inactivation risks, and enhance monitoring in vulnerable populations to ensure safe clinical translation.

Finally, the integrated “therapy-delivery” strategy using natural carrier materials shows significant translational potential. Some natural active ingredients (e.g., chitosan, hyaluronic acid) not only possess inherent anti-inflammatory and mucosal repair activities but can also serve as efficient delivery carriers to enhance the retention and bioavailability of 5-ASA at inflammatory sites, achieving dual functions of therapy and delivery (Jhundoo et al., 2020; Jhundoo et al., 2021a). Recent studies further indicate that environmentally responsive delivery systems based on natural materials such as chitosan, pectin, and alginate, constructed via nanotechnology and microfluidic processes, can significantly improve the colon-targeting ability and local efficacy of mesalazine (Ahmed Najar et al., 2024; Ostovar et al., 2025). Future efforts should further explore novel natural carriers with combined therapeutic and delivery functions and promote the clinical translation of intelligent delivery systems to achieve precision, enhanced efficacy, and reduced toxicity in combination therapies.

## 4 Discussion

The combination of 5-ASA with TCM and its bioactive constituents represents an important advance in the therapeutic strategy for ulcerative colitis. By integrating the strengths of traditional and Western medicine, this approach achieves synergistic efficacy through coordinated actions across multiple physiological levels. At the core of this synergy is the direct inhibition of key inflammatory pathways, such as NF-κB and MAPK, which alleviates intestinal immune dysregulation and curtails excessive tissue damage. Beyond this primary anti-inflammatory effect, a cascade of secondary regulatory processes further support disease control. These include reinforcement of intestinal epithelial barrier integroty, the modulation of gut microbiota composition and metabolic profiles, and improvement of antioxidant capacity, all of which contribute to mucosal repair and sustained disease stability. In addition, certain natural components, such as hyaluronic acid and chitosan, enhance drug delivery and mucosal adhesion, introducing a pharmacokinetic dimension of synergy that improves the local bioavailability of 5-ASA at inflammatory sites.

Through this multi-layered and multi-target mode of action, combination therapy not only controls local intestinal inflammation but also exerts broader regulatory effects on immune balance, microbial homeostasis, and microcirculation, thereby addressing limitations of monotherapy, including incomplete efficacy and poor durability of response. Clinically, this strategy is associated with improved remission rates, endoscopic outcomes, and mucosal healing, as well as relief of core symptoms and extraintestinal manifestations, without an increased risk of adverse effects, consistent with an “efficacy-enhancing and toxicity-reducing” profile. Nevertheless, several challenges remain, including the need for deeper mechanistic clarification, high-quality multicenter clinical evidence, systematic evaluation of long-term safety and drug interactions, and standardized criteria for personalized treatment. Future research should therefore integrate multi-omics approaches, network pharmacology, and artificial intelligence to elucidate synergistic mechanisms, advance large-scale clinical trials, and promote the development of intelligent, natural-source delivery systems to support the precision and broader application of combination therapy.

## Glossary

5-ASA: 5-aminosalicylic acid
UC: ulcerative colitis
TCM: traditional Chinese medicine
NF-κB: nuclear factor kappa-light-chain-enhancer of activated B cells
MAPK: mitogen-activated protein kinase
ERK: extracellular signal-regulated kinase
TNF-α: tumor necrosis factor-alpha
IL: interleukin (e.g., IL-1β, IL-4, IL-6, IL-8, IL-10)
CRP: C-reactive protein
Hs-CRP: high-sensitivity C-reactive protein
DAI: disease activity index
MHC-II: major histocompatibility complex class II
STAT: signal transducer and activator of transcription (e.g., STAT3, STAT6)
Th: T helper cell (e.g., Th1, Th2)
CB: cannabinoid receptor (e.g., CB1, CB2)
GPR35: G protein-coupled receptor 35
sIgA: secretory immunoglobulin A
SCFAs: short-chain fatty acids
MUC-2: mucin 2
ZO-1: zonula occludens-1
TLR: Toll-like receptor
MDA: malondialdehyde
SOD: superoxide dismutase
GPx: glutathione peroxidase
GSH: glutathione
ROS: reactive oxygen species
MPO: myeloperoxidase
LPS: lipopolysaccharide
MMP-9: matrix metalloproteinase-9
eNOS: endothelial nitric oxide synthase
VEGF: vascular endothelial growth factor
PLT: platelets
MPV: mean platelet volume
IPA: indole-3-propionic acid
IAA: indole-3-acetic acid
AhR: aryl hydrocarbon receptor
PXR: pregnane X receptor
BDNF: brain-derived neurotrophic factor
TrkB: tropomyosin receptor kinase B
p75NTR: p75 neurotrophin receptor
RCT: randomized controlled trial
GI: gastrointestinal
